# Logistic and time burdens reported by gynecologic and breast cancer survivors

**DOI:** 10.1186/s12905-025-03826-9

**Published:** 2025-06-02

**Authors:** Allison C. Dona, Aisha Ambo, Patricia Jewett, Katherine Brown, Helen Parsons, Arjun Gupta, Deanna Teoh, Anne Blaes, Rachel I. Vogel

**Affiliations:** 1 Department of Obstetrics, Gynecology, and Women’s Health, University of Minnesota, 420 Delaware Street SE MMC 395, Minneapolis, MN 55455 USA; 2 Division of Epidemiology and Community Health, University of Minnesota, 1300 S 2nd St UNIT 300, Minneapolis, MN 55454 USA; 3 School of Medicine, University of Minnesota, 420 Delaware Street SE, Mayo Building, Minneapolis, MN 55455 USA; 4 Division of Health Policy and Management, University of Minnesota, 420 Delaware St. SE MMC 729, Minneapolis, MN 55455 USA; 5 Division of Hematology, Oncology and Transplantation, University of Minnesota, 420 Delaware Street SE MMC 480, Minneapolis, MN 55455 USA

**Keywords:** Breast cancer, Gynecologic cancer, Logistic burden, Treatment burden, Survivorship, Quality of life

## Abstract

**Background:**

We assessed the effect of the time requirements of cancer care on other life activities and time burden reduction priorities among breast and gynecologic cancer survivors.

**Methods:**

A total of 224 participants with gynecologic or breast cancer from two cohort studies completed a cross-sectional survey regarding logistic and time burdens of cancer care. We compared agreement with the importance of minimizing travel time, wait time, and trips to the cancer center as well as whether cancer care visits interfered with other activities (dependent care, chores, leisure activities) by employment, education, cancer type, cancer treatment status, dependent status, rural-urban residence, and income.

**Results:**

About half (108/217, 49.8%) of participants agreed minimizing time burdens was important. Some agreed that cancer care visits interfered with leisure time (31/215, 14.4%) or impacted their ability to care for dependents (17/215, 7.9% [15.9% among those with dependents, 7/44]). Retired participants, compared to working participants, less often agreed with the importance of minimizing travel time (36.5% [31/85] versus 58.0% [58/100]), trips to the cancer center (34.5% [30/87] versus 58.0% [58/100]), and wait time (35.8% [29/81] versus 56.0% [56/100]). Participants with incomes <$50,000 and those receiving maintenance treatment most often agreed that minimizing wait time was important. Those not working (and not retired or on disability) and those receiving active treatment most often agreed that care interfered with leisure activities.

**Conclusions:**

Minimizing the time needed for cancer care-related tasks matters to patients, especially to those with lower incomes, receiving treatment, and working. In-depth research among demographically diverse populations is needed to evaluate specific time use patterns within cancer care and their association with objective and subjective burden.

**Supplementary Information:**

The online version contains supplementary material available at 10.1186/s12905-025-03826-9.

## Introduction

Improvements in cancer treatments, early detection, and longer life expectancies have led to an increase in the number of cancer survivors. In 2022, the number of cancer survivors reached 18.1 million in the United States (US) and is predicted to grow to 22.5 million by 2032 [1]. Breast and gynecologic cancer survivors make up 22% and 8% of all cancer survivors, respectively [1, 2].

Given the growing number of individuals living with and beyond a cancer diagnosis, understanding the everyday experiences of cancer survivors is increasingly important. One active area of investigation is the “treatment burden” of cancer care. In chronic disease management, treatment burden is considered to be the work of being a patient: the actions and resources required for healthcare tasks and their effects on patient functioning and wellbeing [3]. Consequences of treatment burden in chronic disease include poor health and wellbeing, ineffective resource use, and burden on partners [4].

One aspect of cancer treatment burden progressively studied over the past decade is “financial toxicity”: the cumulative financial impact of cancer and its treatments on patients [5]. Cancer-related costs have been increasing for decades [6]. In a study of 9.5 million new cancer survivors, 42.4% had spent their entire life savings after two years of treatment [7]. In addition to material costs, many cancer survivors experience emotional distress due to financial strain [8]. Risk factors for cancer-related financial toxicity include low income, being single, having dependents, lower education, compromised employment status, female gender, younger age, and belonging to a racialized group, i.e. a group that often encounters systemic barriers due to societal racial biases [7, 9–11]. Financial toxicity has been investigated specifically in people with breast and gynecologic cancers; in breast cancer, for example, financial toxicity has been shown to impact patients across several countries, have both long- and short- term effects, and impact breast cancer-specific care such as reconstruction surgery and lymphedema decision-making [12–16]. Financial toxicity is now widely acknowledged in the cancer community, even though solutions remain mostly elusive.

In contrast to financial toxicity, other aspects of cancer treatment burden, including logistic burdens (also known as logistic toxicity) and time burdens (also known as time toxicity), have been understudied. While these constructs can overlap, time and logistic burdens are distinct from financial toxicity [17, 18]. We define time burdens as time spent on cancer care and associated activities (traveling to appointments, waiting time, etc.) that interferes with a patient’s other life responsibilities and well-being [19–22]. Similarly, we define logistic burdens as patients’ struggle to balance everyday life activities with cancer care (scheduling appointments, pharmacy visits, insurance paperwork, managing drugs, etc.) [23]. This field of study is in its infancy, but similar to financial toxicity, effects of time and logistic burdens are likely objective (actual time spent on cancer activities, number of cancer-related tasks), intersectional (time opportunity costs, loss of time and independence depending on one’s individual circumstances), relational (strained social and family relationships, caregiver burden), and subjective (stress, anger, anxiety, worry) [17, 24]. Loved ones and support persons can be affected by time and logistic burdens of cancer; for example, many patients require caregivers to attend clinic visits for treatment or side effect management [25]. Drawing parallels with financial toxicity, logistic and time burdens may lead to worse outcomes [4, 26–31] and likely reinforce systemic burdens for disadvantaged patients, such as people with low-incomes and populations of color [32, 33].

Recorded objective components of logistic and time burdens (e.g. number of appointments, number of inpatient days, wait time, travel time) have begun to be compared by clinical factors in breast and other cancers [22, 34–36], and qualitative interviews have described logistic and time objective and subjective burdens from patient perspectives [17]. However, the proportion of individuals negatively impacted by the time and logistic requirements of cancer care and that view reducing these burdens as a priority is less clear. Our aim in this study was to quantify the importance of minimizing time spent on cancer care related tasks and whether cancer care visits interfered with other life activities among breast and gynecologic cancer survivors. We also sought to describe clinical and demographic factors associated with greater burden. We hypothesized that reducing time and logistic burdens would be a priority among participants, interfere with participants’ other life tasks, and be most frequently reported by select patient groups, including those who are working, have dependents, report a lower annual household income, are receiving treatment for initial diagnosis or progression, and live in rural areas. This study was a first step in quantifying time burdens, beyond just time itself, and designed to support ongoing and future research in this area.

## Methods

### Population, design, and data collection

The cross-sectional data analyzed in this study came from two prospective cohort survey studies of patients with gynecologic and breast cancer receiving care within the University of Minnesota/ M Health Fairview system. Both studies’ methods are similar and have been previously described [37, 38]. Briefly, The Gynecologic Oncology - Life after Diagnosis (GOLD) study recruited 457 English-speaking gynecologic cancer (cervix, ovary, uterine, vaginal, vulvar) survivors between 2017 and 2020 who were aged 18 years or older and received care within the system prior to study entry. The Breast Oncology - Life after Diagnosis (BOLD) study recruited 217 breast cancer patients between 2018 and 2020 with similar criteria. The purpose of the GOLD and BOLD studies was to describe breast and gynecologic cancer survivorship issues with the goal of supporting interventions focused on optimizing outcomes and quality of life. Individuals were recruited in-person and by mail and were eligible regardless of time since initial diagnosis. In 2022, participants from both cohort studies who were still alive and participating in the cohorts (286 in GOLD and 197 in BOLD) were sent a cross-sectional survey, via email or mail depending on participant preference, designed to examine logistic and time burdens in these survivor populations; the data were pooled for this analysis. Both studies were approved by the University of Minnesota Institutional Review Board (GOLD: 161201581, BOLD: STUDY00002747). All participants provided written informed consent.

### Measures

The primary outcomes of interest were self-reported time and logistic burdens, measured with the Oncology Opportunity Cost Assessment Tool (OOCAT). The full questionnaire was developed by others and has been published previously, with further validation studies in progress [39]. The OOCAT was developed using a patient engaged approach through focus groups and interviews, expanding and refining a list of opportunity cost concepts. The final OOCAT consists of 18 items examining appointment time and the logistical, financial, and quality of life implications of traveling to appointments with multiple item types [39]. We focused our analysis specifically on items asking about the priorities and quality of life impacts of time and logistic requirements of cancer care. These included agreement with “Minimizing wait time (or travel time, trips to the cancer center) is important to me”, “Cancer center visits interfere with my time enjoying my hobbies or other leisure activities,” “Cancer center visits impact my ability to care for dependents (children, family members, friends, pets),” and “Cancer center visits interfere with my time spent taking care of responsibilities like cooking dinner or other daily chores”). We dichotomized the 7-level Likert scale answers into agree (somewhat agree, agree, strongly agree) versus disagree (strongly disagree, disagree, somewhat disagree, neither disagree nor agree).

Primary exposures of interest were employment status (working [full- or part-time] / on disability [short- or long- term]/ retired/ not currently working [e.g. stay-at-house partner or currently unemployed]), whether they report having dependents (yes/no), annual household income (<$50,000/ $50,000–99,999/ ≥$100,000/ prefer not to say), residential status (urban/rural: defined by Zip-code level Rural-Urban Commuting Area [RUCA] codes, categorization C) [40], education status (no college degree/ at least a college degree), treatment status (not receiving treatment/ receiving treatment for initial diagnosis or progression/ maintenance [longer-term treatments to delay or prevent cancer recurrence e.g. PARP inhibitors or hormone therapy after up-front treatment]), and primary cancer site (ovarian/ cervical/ endometrial/ vaginal or vulvar/ breast). An additional descriptive variable, time since diagnosis, was included and is defined as time since initial diagnosis with gynecologic or breast cancer. All demographic and clinical variables were participant self-reported and updated at the time of the OOCAT survey except gender, race and ethnicity, and rural/urban residence, which were reported at baseline.

### Statistical analysis

Data were summarized using descriptive statistics. We compared survivor priorities and interference with other life tasks by work status, dependent status, annual household income, education, rural-urban status, treatment status, and cancer type using Chi-squared and Fisher’s Exact tests as appropriate. We conducted two supplemental analyses: a sensitivity analysis stratifying all analyses by age group (< 65 years, 65 + years) and multivariate analyses simultaneously examining age, income, treatment status, and work status in select outcomes. We recommend caution when interpreting multivariate results because of insufficient samples sizes to fully disentangle the effects of correlated variables of interest (e.g. working status and income). We focused the multivariate analyses on the outcomes, “Minimizing wait time (or travel time, trips to the cancer center) is important to me” because of variation in outcome distributions; multivariate logistic regression models adjusted for age at survey, income, treatment status (active treatment yes/no), and work status (yes/no, with “no” including those retired, on disability, or not working). Because item response missingness was low (approximately 5%), we omitted individuals from analyses only for those missing the particular characteristic or outcome response (pairwise deletion). Analyses were conducted in SAS 9.4 (Cary, North Carolina).

## Results

Of 483 individuals invited, 252 (52.2%) returned this follow-up survey, with 224 (47.4%; 134/286 = 46.8% in GOLD and 90/197 = 45.7% in BOLD) participants completing the OOCAT questionnaire. Those who did not respond were slightly younger than those who responded (median age 59.1 versus 61.4 years at study entry, *p* = 0.006); however, they did not significantly differ with regard to other demographic or clinical characteristics.

Among participants, the most common cancer was breast cancer (90, 40.2%), followed by endometrial (62, 27.7%), ovarian (45, 20.1%), cervical (17, 7.6%), and vaginal or vulvar (10, 4.5%) cancer (Table 1). Median age at time of this survey was 64.2 years (range: 33.7–82.9 years), and median time since diagnosis was 5.2 years (range: 1.4–27.3 years). The majority (211/223, 94.6%) of participants reported being non-Hispanic White. Most (138/213, 64.8%) participants had a partner, and 45 (21.1%) reported having dependents. The distribution of participants by annual household income was fairly even; 22.1% (47/213) reported less than $50,000, 31.5% (67/213) reported $50,000–99,999, 28.6% (61/213) more than $100,000, and 17.8% (38/213) preferred not to report their income. More than half (59.8%, 125/209) of participants had at least a college degree, and most (89.3%, 200/224) lived in an urban area.

**Table 1:** Study participant demographic and clinical characteristics (*N* = 224)

Characteristic	*n*	%
**Age at survey**,** years**,** median (range)**	64.2 (33.7–82.9)	
**Time since diagnosis at survey**,** years**,** median (range)**	5.2 (1.4–27.3)	
**Self-reported gender at baseline**		
Female	207	99.0
Male	0	0.0
Non-binary	2	1.0
*Missing*	*15*	
**Race/Ethnicity at baseline**		
Hispanic, Multiracial	2	0.9
Hispanic, White	2	0.9
Non-Hispanic Asian	3	1.3
Non-Hispanic Black	2	0.9
Non-Hispanic, Multiracial	3	1.3
Non-Hispanic White	211	94.6
*Missing*	*1*	
**Partner status at survey**		
Single/Divorced/Widow	75	35.2
Partnered/married	138	64.8
*Missing*	*11*	
**Any dependents at survey**		
No	168	78.9
Yes	45	21.1
*Missing*	*11*	
**Annual household income at survey**		
<$50,000	47	22.1
$50,000–99,999	67	31.5
≥$100,000	61	28.6
Prefer not to say	38	17.8
*Missing*	*11*	
**Highest Education at survey**		
No college degree	84	40.2
At least college degree	125	59.8
*Missing*	*15*	
**Residence at baseline**		
Rural	24	10.7
Urban	200	89.3
**Work status at survey**		
Retired	89	41.8
Working	103	48.4
Disability	9	4.2
Not working	12	5.6
*Missing*	*11*	
**Cancer disease stage at diagnosis**		
Early (0 / I / II)	145	65.3
Advanced (III / IV)	77	34.7
*Missing*	*2*	
**Cancer type at survey**		
Breast	90	40.2
Cervical	17	7.6
Endometrial	62	27.7
Ovarian	45	20.1
Vaginal or Vulvar	10	4.5
**Current treatment status at survey**		
Not receiving treatment	124	58.2
Receiving treatment for initial diagnosis, progression, or recurrence	36	16.9
Maintenance therapy	53	24.9

Approximately half of all participants agreed that minimizing travel time, trips to the cancer center, and wait time was important to them (Table 2). Survivor priorities varied significantly by work status. Although reducing time burdens was valued across groups, those who were retired were least likely to agree with the importance of minimizing travel time (36.5% [31/85] compared with those with a different work status: 56–67% [58/100 working, 5/9 disability, 8/12 not working]; *p* = 0.02), trips to the cancer center (34.5% [30/87] versus 55–58% [58/100 working; 4/9 disability; 7/12 not working]; *p* = 0.01), and wait time (35.8% [29/81] versus 56–67% [56/100 working; 5/9 disability; 8/12 not working]; *p* = 0.02). In addition to work status, outcomes varied by income. Those with an annual household income less than $50,000 were most likely to endorse the importance of minimizing time. The proportion of survivors who agreed that minimizing wait time was important was highest among those with an annual household income less than $50,000 (66.7% [28/42] versus 35–51% in other groups [23/65 $50,000–99,999; 29/59 ≥$100,000; 19/37 prefer not to say]; *p* = 0.02). Outcomes also varied by treatment status; participants receiving maintenance therapy most frequently reported that minimizing wait time was important to them (58.8% [30/51]) compared to those not receiving treatment (44.8% [52/116]) or receiving treatment for initial diagnosis, progression, or recurrence (48.6% [17/35]; *p* = 0.005). We did not observe differences in these outcomes by dependent status, education, rural-urban status, or cancer site.

The sensitivity analyses—first stratified by age < 65 and 65 + years and second a multivariate analysis simultaneously adjusting for age, treatment status, income and working status—generally supported the conclusions of the univariate models, with some differences between age groups (Supplemental Tables 1–3).

Some (31/215, 14.4%) participants agreed that cancer care visits interfered with leisure time activities, 10.2% (22/215) agreed it interfered with daily chores, and 7.9% (17/215) reported interference with their ability to care for their dependents (15.9% among those with dependents, 7/44; Table 3). Reports of whether cancer care interfered with other life activities varied by work status. Specifically, those who were not currently working (but not retired or on disability) more often agreed that cancer care interfered with leisure activities (41.7% [5/12] versus 12–22% [10/86 retired; 12/100 working; 2/9 disability]; *p* = 0.04). Those receiving treatment for initial diagnosis, progression, or recurrence more frequently reported interference with their leisure activities (28.6% [10/35]) compared to those not receiving treatment (11.8% [14/119]) or on maintenance therapy (11.3% [6/53]; *p* = 0.03). We did not observe differences in these outcomes by dependent status, income, education, rural-urban status, or cancer site.

The proportion of participants reporting interference with life tasks were generally similar when stratified by age group, with some exceptions (Supplemental Tables 4 and 5). Cancer center visits interfering with hobbies and leisure activities was most frequently reported among those receiving active treatment, with a larger difference among participants under 65 years (38.9% [7/18] versus not receiving treatment 11.9% [7/59]; over 65 years 17.7% [3/17] versus 11.7% [7/60]). Interference with hobbies and leisure activities among those with household annual incomes <$50,000 was also more frequently reported among those younger than 65 years (36.8% [7/19] versus 16% [4/25] among those aged 65 or older).

**Table 2:** Participant priorities in reducing time and logistic burdens by clinical and demographic characteristics

Characteristic	Minimizing […] is important		
	Travel time, *n* (Total *n*, %)*	Trips to cancer center, *n* (Total *n*, %)*	Wait time, *n* (Total *n*, %)*
**Everyone**	109 (217; 50.2)	103 (216, 47.7)	103 (210, 49.1)
**Work status**	*p* = 0.02	*p* = 0.01	*p* = 0.02
Retired	31 (85, 36.5)	30 (87, 34.5)	29 (81, 35.8)
Working	58 (100, 58.0)	58 (100, 58.0)	56 (100, 56.0)
Disability	5 (9, 55.6)	4 (9, 44.4)	5 (9, 55.6)
Not working	8 (12, 66.7)	7 (12, 58.3)	8 (12, 66.7)
**Dependents**	*p* = 0.40	*p* = 0.38	*p* = 0.24
No	78 (161, 48.5)	75 (163, 46.0)	73 (157, 46.5)
Yes	25 (45, 55.6)	24 (45, 53.3)	26 (45, 57.8)
**Annual household income**	*p* = 0.75	*p* = 0.18	*p* = 0.02
<$50,000	24 (45, 53.3)	28 (46, 60.9)	28 (42, 66.7)
$50,000–99,999	30 (66, 45.5)	31 (66, 47.0)	23 (65, 35.4)
≥$100,000	29 (59, 49.2)	24 (60, 40.0)	29 (59, 49.2)
Prefer not to say	20 (36, 55.6)	16 (37, 43.2)	19 (37, 51.4)
**Residential location**	*p* = 0.84	*p* = 0.50	*p* = 0.24
Rural	11 (21, 52.4)	12 (22, 54.6)	7 (20, 35.0)
Urban	98 (196, 50.0)	91 (194, 46.9)	96 (190, 50.5)
**Education**	*p* = 0.25	*p* = 0.39	*p* = 0.16
No college degree	42 (80, 52.5)	41 (80, 51.3)	32 (76, 42.1)
At least a college degree	54 (122, 44.3)	55 (122, 45.1)	63 (120, 52.5)
**Treatment status**	*p* = 0.45	*p* = 0.28	*p* = 0.005
Not receiving treatment	58 (118, 49.2)	62 (120, 51.7)	52 (116, 44.8)
Receiving treatment for initial diagnosis, progression, or recurrence	21 (35, 60.0)	17 (36, 47.2)	17 (35, 48.6)
Receiving maintenance therapy	25 (53, 47.2)	20 (52, 38.5)	30 (51, 58.8)
**Cancer site**	*p* = 0.96	*p* = 0.84	*p* = 0.21
Ovarian	21 (45, 46.7)	20 (44, 45.5)	19 (44, 43.2)
Cervical	8 (16, 50.0)	7 (16, 43.8)	8 (14, 57.1)
Endometrial	30 (59, 50.9)	28 (59, 47.5)	22 (57, 38.6)
Vaginal/Vulvar	5 (8, 62.5)	6 (9, 66.7)	5 (8, 62.5)
Breast	45 (89, 50.6)	42 (88, 47.7)	49 (87, 56.3)

* Somewhat agreed / agreed / strongly agreed; Denominator for each outcome may not be identical to total N of each exposure category because of missing outcome value

**Table 3:** Cancer center visit impacts on participants’ other life activities

Characteristic	Impacts ability to care for dependents, *n* (Total *n*, %)*	Interferes with hobbies or other leisure activities, *n* (Total *n*, %)*	Interferes with responsibilities / chores, *n* (Total *n*, %)*
**Everyone**	17 (215, 7.9)	31 (215, 14.4)	22 (215, 10.2)
**Work status**	*p* = 0.14	*p* = 0.04	*p* = 0.07
Retired	6 (86, 7.0)	10 (86, 11.6)	7 (86, 8.1)
Working	7 (100, 7.0)	12 (100, 12.0)	9 (100, 9.0)
Disability	1 (9, 11.1)	2 (9, 22.2)	1 (9, 11.1)
Not working	3 (12, 25.0)	5 (12, 41.7)	4 (12, 33.3)
**Dependents**	*NA*	*p* = 0.76	*p* = 0.40
No	NA	23 (163, 14.1)	15 (163, 9.2)
Yes	6 (44, 13.6)	7 (44, 15.9)	6 (44, 13.6)
**Annual household income**	*p* = 0.25	*p* = 0.13	*p* = 0.29
<$50,000	7 (44, 15.9)	11 (44, 25.0)	8 (44, 18.2)
$50,000–99,999	5 (66, 7.6)	8 (66, 12.1)	6 (66, 9.1)
≥$100,000	3 (61, 4.9)	6 (61, 9.8)	4 (61, 6.6)
Prefer not to say	2 (36, 5.6)	4 (36, 11.1)	3 (36, 8.3)
**Residential location, total N = 224**	*p* = 0.38	*p* = 0.75	*p* = 0.70
Rural	0 (21, 0.0)	2 (21, 9.5)	1 (21, 4.8)
Urban	17 (194, 8.8)	29 (194, 15.0)	21 (194, 10.8)
**Education**	*p* = 0.91	*p* = 0.96	*p* = 0.55
No college degree	6 (78, 7.7)	11 (78, 14.1)	9 (78, 11.5)
At least a college degree	10 (123, 8.1)	17 (123, 13.8)	11 (123, 8.9)
**Treatment status**	*p* = 0.07	*p* = 0.03	*p* = 0.59
Not receiving treatment	14 (119, 11.8)	14 (119, 11.8)	12 (119, 10.1)
Receiving treatment for initial diagnosis, progression, or recurrence	2 (35, 5.7)	10 (35, 28.6)	5 (35, 14.3)
Receiving maintenance therapy	1 (53, 1.9)	6 (53, 11.3)	4 (53, 7.6)
**Cancer site**	*p* = 0.27	*p* = 0.25	*p* = 0.34
Ovarian	5 (44, 11.4)	8 (44, 18.2)	6 (44, 13.6)
Cervical	1 (16, 6.3)	3 (16, 18.8)	3 (16, 18.8)
Endometrial	4 (58, 6.9)	4 (58, 6.9)	3 (58, 5.2)
Vaginal/Vulvar	2 (8, 25.0)	2 (8, 25.0)	1 (8, 12.5)
Breast	5 (89, 5.6)	14 (89, 15.7)	9 (89, 10.1)

* Somewhat agreed / agreed / strongly agreed; Denominator for each outcome may not be identical to total N of each exposure category because of missing outcome value

## Discussion

In the current study, we highlight priorities for and differences in importance of minimizing time and logistic burdens among gynecologic and breast cancer survivors. In addition to quantifying the amount of time patients spend receiving cancer care, it is important to evaluate if and how much reducing that time matters to patients and how those time requirements are impacting their lives. Further, we must identify groups most impacted by time and logistic burdens to create meaningful targeted interventions. This study highlights variation in responses and provides preliminary evidence of demographic and clinical groups most at risk of being burdened by time and logistic demands of cancer care, including those of working age, with lower incomes, and those receiving active or maintenance treatment. These results are hypothesis generating, as further research is needed to understand the interplay between age, working status, and income.

We found that those who worked, were on disability, or did not currently work (but not retired or on disability) were more likely to agree that minimizing their travel time, trips to the cancer center, and wait time was important, compared to those who were retired. This finding may be related to patients having more time and flexibility during retirement and that patients of working age may be most affected by time and logistic burdens of cancer, pointing to the need for more in-depth research among this group [41]. Those who were not currently working agreed that cancer care interfered with leisure activities most frequently, consistent with previously observed associations between unemployment and treatment burden in chronic disease [4].

The results regarding income and logistic and time burdens were mixed and varied by age. Previous research on financial toxicity in gynecologic cancer survivors observed that lower income and unemployment were associated with high financial toxicity [10]. Time and financial burdens have been observed to compound one another [17], such as when patients have to miss work to treat side effects of cancer treatment [25]. Further research is needed to understand how financial, time, and logistic cancer burdens may intersect and interact with each other.

In addition to differences by socioeconomic variables, time and logistic burdens varied by treatment status. Those receiving maintenance therapy more frequently reported that minimizing wait time was important than those currently receiving active treatment or no treatment. As expected, those receiving active treatment more frequently reported that their cancer treatment interfered with leisure activities than those receiving maintenance therapy or no treatment, especially among those less than 65 years of age. Greater time and logistic burdens would generally be hypothesized for those in active treatment given the greater number of appointments and new changes related to care; however, our finding that those in maintenance treatment are also affected highlights the importance of investigating the potential effects of chronic cancer time burdens. Our findings further suggest that participant priorities and perceptions of the impact of the burden matter in addition to the amount of time itself, emphasizing that measuring objective time does not necessarily equate to an understanding of time burden. Additionally, while it is a strength to capture experiences of cancer survivors who both are and are not receiving active treatment, it is also difficult to combine these groups when their priorities may differ. Future research should attempt to confirm these results and investigate this relationship in other cancer types, as medications used for maintenance therapies and the context with which they are used varies.

More frequent prioritization of minimizing time and logistic burdens of cancer care among those with additional life challenges and time commitments aligns with chronic disease models of cumulative patient complexity and workload-capacity imbalance. When clinical and social factors accumulate, workload expands, and capacity to handle that workload decreases, which disrupts access, utilization, self-care, and health [42]. If patients with additional time commitments and stressors are expending their energy and cognitive resources needed for self-management elsewhere, they would likely prioritize minimizing time burdens of their care more than other groups, as observed in our study.

A strength of this study is its focus on gynecologic and breast cancer survivors, who may experience more time burden than other cancer survivors. One study found that individuals with ovarian cancer had the highest initial care time burdens of 12 cancers studied, and individuals with breast cancer were also spending more time on cancer care than individuals with other cancers [43]. Women may also have many competing time burdens; for example, despite changes in societal gender roles, unpaid caregiving responsibilities, often in addition to paid work, remain more common among women [4]. Our study also serves as a step towards describing intersections of treatment burden with identity (specifically income, education level, work status, residential location, and dependent status), a gap in knowledge identified by previous scoping review of treatment burden literature in chronic disease [41]. Another strength in this study is the use of the OOCAT tool, asking specifically about how cancer center visits interfere with activities and how minimizing time burdens related to the cancer center are important. This adds to the existing literature, which has quantified objective time burdens (e.g. hours spent on care) in specific cancer settings [22, 34–36] and results from a qualitative study that described time and logistic burdens in detail but could not quantify these burdens or make conclusions specific to people with breast and gynecologic cancers [17].

This work highlights priorities and generates hypotheses for future time and logistic burden research in cancer care. While the OOCAT tool used here has provided new insight into cancer center trip burdens, this tool may underestimate the total time burden of cancer care because these questions do not include other pieces of cancer care that take time (e.g. other medical visits for treatment related side effects, scheduling appointments and sending medical messages from home, etc.). Future work should more comprehensively investigate care tasks that could be streamlined to reduce care time. Additionally, as this work is only reflective of one health system, research is needed to investigate these time burden priorities within other health systems in order to better understand to what extent interventions may be broadly applied or must be tailored to each individual care system. Given variation in policies, procedures, and patient schedules, there are likely differences by institution in logistic burdens.

In addition to investigating which specific burden reduction strategies may work by health system, future research should investigate who is most burdened and may benefit from specific interventions. While we describe differences in burden prioritization and impact by work status, income, and treatment status, both generalizability and subgroup results are limited by small sample sizes, particularly among those on disability or not working, rural residents, and persons of color. Options to run multivariate analyses in this study were limited due to the sample size. However, a key purpose of this study was to generate hypotheses to guide future work in this topic, including more thorough and appropriately powered multivariate analyses to allow for a more clear and accurate understanding of the associations between clinical and social risk factors and time and logistic burdens of cancer care. Given that 94.6% of participants identified as non-Hispanic White, and we were not powered to assess for differences by race and ethnicity, we are likely missing disparities in these groups. Previous work not specific to cancer care has observed greater use of public transportation to receive care among low-income and Black and Hispanic individuals, which may make navigating cancer care more time consuming and impactful on other life activities [44]. Further, the survey response rate in this study may limit the generalizability of the results if those missing have different experiences of time and logistic burdens.

As we more comprehensively understand care burdens and their impacts on patients, research should move toward intervention science, quality improvement, and clinical trials as appropriate to reduce time burdens for people with cancer. Many participants in this study agreed that minimizing time and logistic burdens of cancer care is a priority, which suggests that logistic and time burdens are important patient considerations when planning cancer care. In clinical practice, such considerations are limited given the competing treatment and other care coordination responsibilities for providers. Optimizing cancer care is complex, and time and logistic burdens vary by cancer type, stage, and treatments received [35]. While we confirmed that reducing time burdens mattered to cancer patients, and provided some evidence for whom these burdens matter the most, we did not measure what improvements to clinical practice patients would like to see implemented. Depending on cancer patient priorities, care needs, and resources, potential improvements could include expansion of telemedicine, streamlined clinic workflow, patient navigation, homecare, and/or clinical coordinators [45–47]. Interventions are likely to be complex, involving multiple stakeholders. Of note, potential interventions should be thoughtfully structured to avoid exacerbating existing healthcare inequities, such as telemedicine barriers for patients who experience low income, low health literacy, rural environments, systemic racism, or limited English literacy [48]. This is especially relevant considering our findings suggest that individuals with low income and unstable work situations may face disproportionate impacts of time and logistic care burdens.

## Conclusions

Many breast and gynecologic cancer survivors in this study agreed that minimizing time and logistic burdens is important. The proportion of those who agreed varied by employment status, household income, and cancer treatment status, pointing to sources of potential differences in impact that should be confirmed by in-depth research in order to inform interventions to address these burdens. Future studies should also assess in greater depth which specific care-related tasks (e.g. traveling, waiting times, times with provider, labs, infusions, radiation treatment, etc.) are most time consuming and disruptive and which clinical process flows may lead to an inefficient use of patients’ time. Additional research should also assess interactions between individual circumstances to understand when objective time burdens become subjectively burdensome; for example, the unique experiences of people with low incomes, rural residents, persons of color, and other demographic groups that may experience additional demands on their time.

## Electronic supplementary material

Below is the link to the electronic supplementary material.

Supplementary Material 1

## Data Availability

De-identified data will be available upon email request to the corresponding author (Rachel I. Vogel, isak0023@umn.edu) and ethics board approval.

## Abbreviations

US: United States
(GOLD) study: The Gynecologic Oncology - Life after Diagnosis
(BOLD) study: The Breast Oncology - Life after Diagnosis
OOCAT: Oncology Opportunity Cost Assessment Tool
PARP: Poly(ADP-ribose) polymerase
RUCA: Rural-Urban Commuting Area
