# Supplementary material for: Logistic and time burdens reported by gynecologic and breast cancer survivors
Source: BMC Womens Health. 2025 Jun 2;25:273. doi: 10.1186/s12905-025-03826-9 (PMC12128304; doi:10.1186/s12905-025-03826-9)
Supplement: Supplementary file 1 — Supplementary Material 1 [file 12905_2025_3826_MOESM1_ESM.docx]

**Supplemental Table 1. Participant priorities in reducing time and logistic burdens by clinical and demographic characteristics among participants aged < 65 years old.**

| **Characteristic** | **Minimizing […] is important** | | |
| --- | --- | --- | --- |
|  | **Travel**  **time,**  **N (Total N, %)*** | **Trips to**  **cancer center,**  **N (Total N, %)*** | **Wait**  **time,**  **N (Total N, %)*** |
| **Everyone <65 years old** | 60 (116, 51.7) | 61 (117, 52.1) | 65 (117, 55.6) |
| **Work status** |  |  |  |
| Retired | 5 (15, 33.3) | 6 (15, 40.0) | 7 (15, 46.7) |
| Working | 44 (80, 55.0) | 45 (81, 55.6) | 46 (81, 56.8) |
| Disability | 5 (8, 62.5) | 4 (8, 50.0) | 5 (8, 62.5) |
| Not working | 3 (7, 42.9) | 3 (7, 42.9) | 3 (7, 42.9) |
| **Dependents** |  |  |  |
| No | 38 (73, 52.1) | 38 (74, 51.4) | 39 (74, 52.7) |
| Yes | 21 (39, 53.9) | 21 (39, 53.9) | 23 (39, 59.0) |
| **Annual household income** |  |  |  |
| <$50,000 | 9 (18, 50.0) | 13 (19, 68.4) | 13 (18, 72.2) |
| $50,000-99,999 | 15 (32, 46.9) | 17 (32, 53.1) | 12 (32, 37.5) |
| ≥$100,000 | 21 (42, 50.0) | 20 (43, 46.5) | 23 (43, 53.5) |
| Prefer not to say | 14 (19, 73.7) | 9 (19, 47.4) | 14 (20, 70.0) |
| **Residential location** |  |  |  |
| Rural | 1 (4, 25.0) | 2 (4, 50.0) | 1 (4, 25.0) |
| Urban | 59 (112, 52.7) | 59 (113, 52.2) | 64 (116, 56.6) |
| **Education** |  |  |  |
| No college degree | 18 (35, 51.4) | 20 (35, 57.1) | 16 (35, 45.7) |
| At least a college degree | 34 (72, 47.2) | 37 (73, 50.7) | 44 (73, 60.3) |
| **Treatment status** |  |  |  |
| Not receiving treatment | 29 (57, 50.9) | 34 (59, 57.6) | 29 (59, 49.2) |
| Receiving treatment for initial  diagnosis, progression, or recurrence | 12 (18, 66.7) | 10 (18, 55.6) | 10 (18, 55.6) |
| Receiving maintenance therapy | 18 (37, 48.7) | 15 (36, 41.7) | 23 (36, 63.9) |
| **Cancer site** |  |  |  |
| Ovarian | 9 (19, 47.4) | 8 (19, 42.1) | 7 (19, 36.8) |
| Cervical | 7 (12, 58.3) | 5 (12, 41.7) | 7 (11, 63.6) |
| Endometrial | 10 (25, 40.0) | 13 (25, 52.0) | 10 (26, 38.5) |
| Vaginal/Vulvar | 2 (4, 50.0) | 4 (5, 80.0) | 3 (5, 60.0) |
| Breast | 32 (56, 57.1) | 31 (56, 55.4) | 38 (56, 67.9) |

* Somewhat agreed / agreed / strongly agreed; Denominator for each outcome may not be identical to total N of each exposure category because of missing outcome value

**Supplemental Table 2. Participant priorities in reducing time and logistic burdens by clinical and demographic characteristics among participants aged 65 years or older.**

| **Characteristic** | **Minimizing […] is important** | | |
| --- | --- | --- | --- |
|  | **Travel**  **time,**  **N (Total N, %)*** | **Trips to**  **cancer center,**  **N (Total N, %)*** | **Wait**  **time,**  **N (Total N, %)*** |
| **Everyone 65 years or older** | 49 (101, 48.5) | 42 (99, 42.4) | 38 (93, 40.9) |
| **Work status** |  |  |  |
| Retired | 26 (70, 37.1) | 24 (72, 33.3) | 22 (66, 33.3) |
| Working | 14 (20, 70.0) | 13 (19, 68.4) | 10 (19, 52.6) |
| Disability | 0 (1, 0.0) | 0 (1, 0.0) | 0 (1, 0.0) |
| Not working | 5 (5, 100.0) | 4 (5, 80.0) | 5 (5, 100.0) |
| **Dependents** |  |  |  |
| No | 40 (88, 45.5) | 37 (89, 41.6) | 34 (83, 41.0) |
| Yes | 4 (6, 66.7) | 3 (6, 50.0) | 3 (6, 50.0) |
| **Annual household income** |  |  |  |
| <$50,000 | 15 (27, 55.6) | 15 (27, 55.6) | 15 (24, 62.5) |
| $50,000-99,999 | 15 (34, 44.1) | 14 (34, 41.2) | 11 (33, 33.3) |
| ≥$100,000 | 8 (17, 47.1) | 4 (17, 23.5) | 6 (16, 37.5) |
| Prefer not to say | 6 (17, 35.3) | 7 (18, 38.9) | 5 (17, 29.4) |
| **Residential location** |  |  |  |
| Rural | 10 (17, 58.8) | 10 (18, 55.6) | 6 (16, 37.5) |
| Urban | 39 (84, 46.4) | 32 (81, 39.5) | 32 (77, 41.6) |
| **Education** |  |  |  |
| No college degree | 24 (45, 53.3) | 21 (45, 46.7) | 16 (41, 39.0) |
| At least a college degree | 20 (50, 40.0) | 18 (49, 36.7) | 19 (47, 40.4) |
| **Treatment status** |  |  |  |
| Not receiving treatment | 29 (61, 47.5) | 28 (61, 45.9) | 23 (57, 40.4) |
| Receiving treatment for initial  diagnosis, progression, or recurrence | 9 (17, 52.9) | 7 (18, 38.9) | 7 (17, 41.2) |
| Receiving maintenance therapy | 7 (16, 43.8) | 5 (16, 31.3) | 7 (15, 46.7) |
| **Cancer site** |  |  |  |
| Ovarian | 12 (26, 46.2) | 12 (25, 48.0) | 12 (25, 48.0) |
| Cervical | 1 (4, 25.0) | 2 (4, 50.0) | 1 (3, 33.3) |
| Endometrial | 20 (34, 58.8) | 15 (34, 44.1) | 12 (31, 38.7) |
| Vaginal/Vulvar | 3 (4, 75.0) | 2 (4, 50.0) | 2 (3, 66.7) |
| Breast | 13 (33, 39.4) | 11 (32, 34.4) | 11 (31, 35.5) |

* Somewhat agreed / agreed / strongly agreed; Denominator for each outcome may not be identical to total N of each exposure category because of missing outcome value

**Supplemental Table 3. Multivariate association of participants characteristics with reducing time and logistic burdens, across everyone and stratified by age, odds ratios (OR) and confidence intervals (CI).**

| **Characteristic** | **Minimizing […] is important** | | | | | |
| --- | --- | --- | --- | --- | --- | --- |
|  | **Travel time** | | **Trips to  cancer center** | | **Wait  time** | |
|  | **OR (95% CI)** | **P** | **OR (95% CI)** | **P** | **OR (95% CI)** | **P** |
| Age at survey, per 5 years | 0.96 (0.80-1.15) | 0.66 | 0.95 (0.79-1.15) | 0.60 | 0.84 (0.69-1.01) | 0.07 |
| Household income, per $10,000 | 0.98 (0.93-1.03) | 0.43 | 0.95 (0.90-1.00) | 0.05 | 0.97 (0.92-1.02) | 0.22 |
| Active treatment, yes versus no | 1.46 (0.61-3.53) | 0.39 | 1.08 (0.46-2.56) | 0.86 | 1.35 (0.56-3.26) | 0.51 |
| Working, yes versus no | 1.77 (0.83-3.77) | 0.14 | 1.82 (0.83-3.96) | 0.13 | 1.08 (0.50-2.34) | 0.85 |
| **Age < 65 years** | | | | | | |
| Age at survey, per 5 years | 0.85 (0.63-1.15) | 0.30 | 1.19 (0.89-1.60) | 0.25 | 0.84 (0.62-1.13) | 0.24 |
| Household income, per $10,000 | 1.01 (0.95-1.07) | 0.87 | 0.97 (0.91-1.03) | 0.32 | 1.00 (0.94-1.06) | 0.94 |
| Active treatment, yes versus no | 1.05 (0.29-3.81) | 0.94 | 1.56 (0.43-5.68) | 0.50 | 1.17 (0.33-4.18) | 0.81 |
| Working, yes versus no | 2.11 (0.73-6.09) | 0.17 | 1.85 (0.66-5.20) | 0.24 | 0.93 (0.34-2.56) | 0.89 |
| **Age 65 years or older** | | | | | | |
| Age at survey, per 5 years | **0.57 (0.33-0.98)** | **0.04** | 0.68 (0.39-1.18) | 0.17 | **0.54 (0.30-0.97)** | **0.04** |
| Household income, per $10,000 | 0.96 (0.87-1.06) | 0.47 | **0.89 (0.79-0.99)** | **0.04** | 0.91 (0.82-1.02) | 0.11 |
| Active treatment, yes versus no | 1.85 (0.49-6.92) | 0.36 | 0.83 (0.24-2.84) | 0.76 | 1.37 (0.37-5.00) | 0.64 |
| Working, yes versus no | 2.48 (0.66-9.32) | 0.18 | 1.48 (0.40-5.49) | 0.56 | 1.71 (0.45-6.61) | 0.43 |

**Supplemental Table 4. Cancer center visits impacts on participants’ other life actives among participants aged < 65 years old.**

| **Characteristic** | **Impacts ability to care for dependents,**  **N (Total N, %)*** | **Interferes with hobbies or other leisure activities,**  **N (Total N, %)*** | **Interferes with responsibilities / chores,**  **N (Total N, %)*** |
| --- | --- | --- | --- |
| **Everyone <65 years old** | 8 (118, 6.8) | 21 (118, 17.8) | 14 (118, 11.9) |
| **Work status** |  |  |  |
| Retired | 0 (16, 0.0) | 4 (16, 25.0) | 2 (16, 12.5) |
| Working | 6 (81, 7.4) | 10 (81, 12.4) | 8 (81, 9.9) |
| Disability | 1 (8, 12.5) | 2 (8, 25.0) | 1 (8, 12.5) |
| Not working | 1 (7, 14.3) | 3 (7, 42.9) | 2 (7, 28.6) |
| **Dependents** |  |  |  |
| No | 3 (76, 4.0) | 14 (76, 18.4) | 8 (76, 10.5) |
| Yes | 5 (38, 13.2) | 6 (38, 15.8) | 5 (38, 13.2) |
| **Annual household income** |  |  |  |
| <$50,000 | 3 (19, 15.8) | 7 (19, 36.8) | 4 (19, 21.1) |
| $50,000-99,999 | 2 (32, 6.3) | 4 (32, 12.5) | 4 (32, 12.5) |
| ≥$100,000 | 2 (43, 4.7) | 5 (43, 11.6) | 3 (4.3, 7.0) |
| Prefer not to say | 1 (19, 5.3) | 3 (19, 15.8) | 2 (19, 10.5) |
| **Residential location** |  |  |  |
| Rural | 0 (5, 0.0) | 1 (5, 20.0) | 0 (5, 0.0) |
| Urban | 8 (113, 7.1) | 20 (113, 17.7) | 14 (113, 12.4) |
| **Education** |  |  |  |
| No college degree | 2 (35, 5.7) | 5 (35, 14.3) | 4 (35, 11.4) |
| At least a college degree | 6 (74, 8.1) | 13 (74, 17.6) | 8 (74, 10.8) |
| **Treatment status** |  |  |  |
| Not receiving treatment | 7 (59, 11.9) | 7 (59, 11.9) | 7 (59, 11.9) |
| Receiving treatment for initial  diagnosis, progression, or recurrence | 0 (18, 0.0) | 7 (18, 38.9) | 2 (18, 11.1) |
| Receiving maintenance therapy | 1 (37, 2.7) | 6 (37, 16.2) | 4 (37, 10.8) |
| **Cancer site** |  |  |  |
| Ovarian | 1 (19, 5.3) | 2 (19, 10.5) | 2 (19, 10.5) |
| Cervical | 0 (12, 0.0) | 2 (12, 16.7) | 2 (12, 16.7) |
| Endometrial | 2 (25, 8.0) | 3 (25, 12.0) | 2 (25, 8.0) |
| Vaginal/Vulvar | 1 (5, 20.0) | 1 (5, 20.0) | 0 (5, 0.0) |
| Breast | 4 (57, 7.0) | 13 (57, 22.8) | 8 (57, 14.0) |

* Somewhat agreed / agreed / strongly agreed; Denominator for each outcome may not be identical to total N of each exposure category because of missing outcome value

**Supplemental Table 5. Cancer center visits impacts on participants’ other life activities among participants aged 65 years and older.**

| **Characteristic** | **Impacts ability to care for dependents,**  **N (Total N, %)*** | **Interferes with hobbies or other leisure activities,**  **N (Total N, %)*** | **Interferes with responsibilities / chores,**  **N (Total N, %)*** |
| --- | --- | --- | --- |
| **Everyone 65 years or older** | 9 (97, 9.3) | 10 (97, 10.3) | 8 (97, 8.3) |
| **Work status** |  |  |  |
| Retired | 6 (70, 8.6) | 6 (70, 8.6) | 5 (70, 7.1) |
| Working | 1 (19, 5.3) | 2 (19, 10.5) | 1 (19, 5.3) |
| Disability | 0 (1, 0.0) | 0 (1, 0.0) | 0 (1, 0.0) |
| Not working | 2 (5, 40.0) | 2 (5, 40.0) | 2 (5, 40.0) |
| **Dependents** |  |  |  |
| No | 8 (87, 9.2) | 9 (87, 10.3) | 7 (87, 8.1) |
| Yes | 1 (6, 16.7) | 1 (6, 16.7) | 1 (6, 16.7) |
| **Annual household income** |  |  |  |
| <$50,000 | 4 (25, 16.0) | 4 (25, 16.0) | 4 (25, 16.0) |
| $50,000-99,999 | 3 (34, 8.8) | 4 (34, 11.8) | 2 (34, 5.9) |
| ≥$100,000 | 1 (18, 5.6) | 1 (18, 5.6) | 1 (18, 5.6) |
| Prefer not to say | 1 (17, 5.9) | 1 (17, 5.9) | 1 (17, 5.9) |
| **Residential location** |  |  |  |
| Rural | 0 (16, 0.0) | 1 (16, 6.3) | 1 (16, 6.3) |
| Urban | 9 (81, 11.1) | 9 (81, 11.1) | 7 (81, 8.6) |
| **Education** |  |  |  |
| No college degree | 4 (43, 9.3) | 6 (43, 14.0) | 5 (43, 11.6) |
| At least a college degree | 4 (49, 8.2) | 4 (49, 8.2) | 3 (49, 6.1) |
| **Treatment status** |  |  |  |
| Not receiving treatment | 7 (60, 11.7) | 7 (60, 11.7) | 5 (60, 8.3) |
| Receiving treatment for initial  diagnosis, progression, or recurrence | 2 (17, 11.8) | 3 (17, 17.7) | 3 (17, 17.7) |
| Receiving maintenance therapy | 0 (16, 0.0) | 0 (16, 0.0) | 0 (16, 0.0) |
| **Cancer site** |  |  |  |
| Ovarian | 4 (25, 16.0) | 6 (25, 24.0) | 4 (25, 16.0) |
| Cervical | 1 (4, 25.0) | 1 (4, 25.0) | 1 (4, 25.0) |
| Endometrial | 2 (33, 6.1) | 1 (33, 3.0) | 1 (33, 3.0) |
| Vaginal/Vulvar | 1 (3, 33.3) | 1 (3, 33.3) | 1 (3, 33.3) |
| Breast | 1 (32, 3.1) | 1 (32, 3.1) | 1 (32, 3.1) |

* Somewhat agreed / agreed / strongly agreed; Denominator for each outcome may not be identical to total N of each exposure category because of missing outcome value
